# Marker of Bone Resorption in Acute Response to Exogenous or Endogenous Parathyroid Hormone

**Published:** 2008-01-25

**Authors:** Vit Zikan, Jan J. Stepan

**Affiliations:** 3rd Department of Internal Medicine and Institute of Rheumatology, Charles University Faculty of Medicine, Prague, Czech Republic

**Keywords:** bone resorption, calcium, C-telopeptide, parathyroid hormone, teriparatide

## Abstract

Parathyroid hormone (PTH) changes morphology of osteoclasts within minutes after its systemic administration. The aim of our study was to test in healthy men whether both exogenous and endogenous PTH could change acutely (minutes to hours) the serum cross-linked C-telopeptide of type I collagen (beta CTX), which is released during osteoclastic resorption of bone. Twelve healthy men (age range 24–34 yr) were each studied during 180 min on a control period, after a single subcutaneous injection of teriparatide, and after 30 min EDTA infusion to stimulate endogenous PTH secretion. The tests were started after overnight fast, 3 h after a standard calcium load. The EDTA infusion induced a significant decrease in serum ionized calcium (by 8.5% at 33 min) and a significant increase in plasma PTH (by 305% at 33 min). Both the EDTA and teriparatide resulted in a significant increase in beta CTX (p < 0.001) with maximum increases of 64% and 80%, respectively. A mild, but significant decrease in beta CTX was observed during the control test period. In conclusion, single-dose teriparatide injection as well as a stimulation of endogenous PTH in healthy men results in an acute increase of the bone resorption marker.

Parathyroid hormone (PTH) increases bone resorption by stimulating both the appearance of new osteoclasts and the activity of mature osteoclasts (Suda et al. 1996; Morony et al. 1999). The effects of PTH on osteoclasts require the participation of stromal cells or osteoblasts which possess high-affinity PTH receptors, although also low-affinity receptors have been identified on osteoclasts (Gay et al. 2003). Experimentally, rapid morphologic changes were documented in preexisting osteoclasts after a single exposure to PTH (Holtrop and King, 1977; Miller, 1978; Holtrop et al. 1979). In young rats, an intravenous injection of PTH resulted in morphological changes of osteoclasts within 30 minutes. These changes coincided with a small increase in plasma calcium concentration (Holtrop et al. 1979), and preceded increase in osteoclast numbers. In humans, the acute (minutes to hours) responses of biochemical markers of bone resorption to a single dose of PTH were not observed by using the urinary hydroxyproline and deoxypyridinoline (Lindsay et al. 1993; Schwietert et al. 1997). Although, in the study of Lindsay et al. on 11 postmenopausal osteoporotic women, a single subcutaneous administration of human PTH (1–34) resulted in a rise of serum calcium concentrations (between 90–240 min), at the same time when serum phosphorus concentrations started to return to normal, despite the continuing increase in a phosphate clearance (Lindsay et al. 1993).

The aim of our study was to test in healthy men whether PTH both exogenous (as a single subcutaneous injection of teriparatide) and endogenous (induced by an acute hypocalcaemia), could change acutely (minutes to hours) the serum concentrations of mature collagen type I degradation fragments (beta CTX) (Rosenquist et al. 1998), the marker which is released into the circulation during osteoclastic resorption of bone.

## Materials and Methods

### Subjects

Twelve healthy men on no medication, mean age 27.7 ± 3.6 years (range 24–34 years), mean height of 184.2 ± 4.9 cm, and mean weight 82.6 ± 9.2 kg participated in this study. The baseline blood pressure, physical examination and electrocardiogram were normal. All subjects were Caucasians and non-smokers. All subjects gave their signed informed consent and the study had been previously approved by the Ethics Committee of the First Medical Faculty, Charles University and General Faculty Hospital, Prague.

### Study design

Twelve men were studied in three sessions 7–14 days apart. The subjects fasted overnight, and for safety reasons and to standardize the baseline concentrations of the variables, all tests were performed 3 h after a standard calcium load (250 mg of elemental calcium in 200 ml of plain water and 30 g of white bread). The maximum decrease in bone resorption occurs approximately 3 hours after such a calcium load (Zikan et al. 2001). All subjects had been instructed to use the standard calcium load at 6:00 and to report time of calcium use.

An intravenous cannula was placed in the forearm and was used for both the EDTA infusion and blood sampling. Blood samples were obtained immediately before the test 3 h after a standard calcium load—approximately at 09:00 (baseline) and exactly at 33, 60, 90, 120 and 180 min after the beginning of the test. The subjects remained fasting until the end of each study session with steady intake of plain water (about 500 ml). The plasma and serum specimens were stored at −70 °C for the later measurement of the bone markers and PTH. Measurement of the serum ionized calcium (iCa) was performed on the same day after anaerobic collection (Vacutainer^®^) of all specimens, which were stored at 4 °C to avoid pH shifts.

The endogenous PTH secretion was stimulated by hypocalcaemia induced by an EDTA infusion. Disodium ethylenediaminetetraacetic acid (EDTA) at a dose of 10 mg/kg body weight in 100 ml 0.9% NaCl solution was administered for 30 min, along with 10–15 ml of 10% trimecaine in 100 ml 0.9% NaCl to avoid the venous irritation and followed by 20 ml 0.9% NaCl alone over 1 min after the end of EDTA infusion.

Human recombinant PTH 1–34, teriparatide (Forsteo^®^, Eli Lilly, Indianapolis, U.S.A.) at a dose of 20 μg was administered as a single injection subcutaneously into the anterior abdominal wall. Only plain water was given after a standard calcium load during the control test period.

### Biochemical analysis

All samples, except the serum ionized calcium, were assayed simultaneously. Serum ionized calcium (iCa) was measured using ion selective electrode with an AVL 9180 (Roche Diagnostics GmbH, Germany). The within run imprecision was below 2% and between run imprecision was below 4%.

The plasma concentrations of the immunoreactive intact PTH were determined using an electrochemiluminesce—based immunoanalysis (the Elecsys 1010 Analyzer, Roche Diagnostics GmbH, Germany). The within run imprecision was below 6%.

The serum concentrations of type I collagen cross-linked C telopeptide (CTX; beta CrossLaps) and N-MID osteocalcin (OC) in the plasma was assessed using the electrochemiluminiscence—based immunoanalysis (the Elecsys 1010 Analyzer, Roche Diagnostics, Germany). The within run imprecision for the CTX was below 7%. The within run imprecision for the OC was below 5%.

The serum concentration of intact aminoterminal propeptide of type I procollagen (PINP) was assessed by radioimmunoassay (Procollagen Intact PINP, Orion Diagnostica, Finland). The within run imprecision was below 5%.

Serum 25-hydroxyvitamin D_3_ (25-OHD_3_) was determined using radioimmunoassay (OCTEIA-25-hydroxy Vitamin D kit, Immunodiagnostic Systems Limited, U.K.) with the normal range of 47.7–144 nmol/l. The between run imprecision was 8.2%.

### Statistical analysis

Data were expressed as mean ± SD if not otherwise stated. The time differences were assessed by one-way ANOVA for repeated measurements. If the treatment effects were not normally distributed, the Friedman repeated measures ANOVA on ranks was used. If a significant difference was found with ANOVA, subsequent multiple comparison procedures (Tukey test) were used to determine the difference between the basal values and other time points. The trapezoidal rule was used to calculate the area under the curve (AUC) for the measured biochemical parameters. The differences between the study sessions were evaluated by the paired t test or ANOVA. Analyses were made with Sigma-Stat statistical software, version 3.1 (Jandel Corporation, San Rafael, U.S.A). The differences are considered significant at p < 0.05.

## Results

The initial (fasting) concentrations of the measured biochemical variables (mean ± 1 SD) were as follows: serum iCa, 1.31 ± 0.02 mmol/l; plasma PTH, 30.3 ± 6 ng/l; serum 25-OHD_3_, 52.8 ± 10 nmol/l; serum beta CTX, 485.6 ± 133 ng/l; plasma osteocalcin, 24.3 ± 6 μg/l; serum PINP, 64.8 ± 17 μg/l. All results were within normal age- and sex- range. All subjects completed the full study protocol with no adverse event.

**Figure 1:**
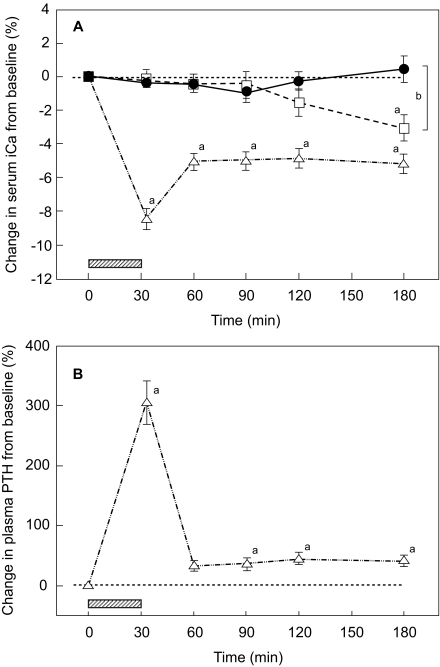
**A.** Mean percentage changes (± SEM) in serum ionized calcium concentrations in response to EDTA infusions (▵) and teriparatide injections (•) in 12 studied men. During the control test period only a standard calcium load was consumed (□). ^a^, p < 0.001 repeated measures ANOVA and p < 0.05 for comparison with baseline after adjustments for multiple comparisons (Tukey test). ^b^, p < 0.05, t-test for comparison AUCs between teriparatide and control test period. **B.** Mean percentage change (± SEM) in plasma intact PTH concentration in response to EDTA infusions in 12 studied men. ^a^, p < 0.001 Friedman repeated measures ANOVA on ranks and p < 0.05 for comparison with baseline after adjustments for multiple comparisons (Dunn’s Method). Hatched area represents EDTA infusion.

Figure 1 shows the mean percentage changes in serum iCa (A) and plasma intact PTH (B) during the control and 2 study periods in all 12 subjects. After the EDTA infusion, there was a significant decrease in serum iCa from a baseline value of 1.32 ± 0.03 mmol/l to 1.21 ± 0.04 mmol/l as early as at 33 min and the decrease remained significant until the end of observation at 180 min (RM ANOVA, p < 0.001; Tukey test, p < 0.05 for all time points versus baseline) (Fig. 1A). No significant changes in serum iCa were detected following teriparatide administration compared to baseline (RM ANOVA, p = 0.235). However, as compared to the control test period, serum iCa was significantly higher at 180 min after teriparatide injection (1.33 ± 0.03 mmol/l and 1.28 ± 0.02 mmol/l, teriparatide and control period, respectively). During the control test period, there was a mild, but significant decrease in serum iCa from a baseline value of 1.32 ± 0.02 to 1.28 ± 0.02 mmol/l at 180 min (RM ANOVA, p < 0.001; Tukey test, p < 0.05).

The hypocalcaemia induced by the infusion of EDTA caused a transient increase in plasma intact PTH from a baseline value of 27 ± 16 ng/l to a peak value of 98 ± 38 ng/l after the end of the 30 min infusion (at 33 min), with subsequent rapid decrease near to the baseline values (Fig. 1B). Concentrations of human PTH 1–34 peptide were not measured in this study. However, a peak of the peptide occurs at about 30 minutes after human PTH 1–34 administration (Lindsay et al. 1993).

**Figure 2:**
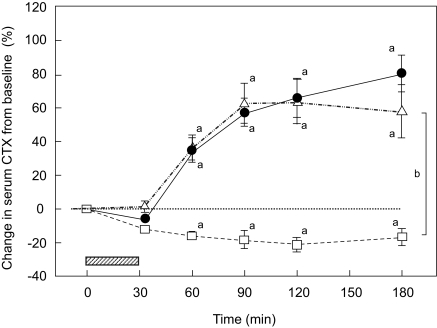
Mean percentage changes (± SEM) in serum beta CTX concentrations in response to EDTA infusions (▵) and teriparatide injections (•) in 12 studied men. During the control test period only a standard calcium load was consumed (□). ^a^, p < 0.001 repeated measures ANOVA and p < 0.05 for comparison with baseline after adjustments for multiple comparisons (Tukey test). ^b^, p < 0.001, ANOVA for comparison AUCs between teriparatide or EDTA and control test period. Hatched area represents EDTA infusion.

Mean changes in the concentrations of serum beta CTX from the beginning to the end of the study periods in all 12 subjects are shown in Figure 2 (mean percentage changes) and in the Table 1 (mean absolute values). The time course for the increase in beta CTX concentrations was highly reproducible in all subjects for both the teriparatide injections and EDTA infusions. Both the EDTA and teriparatide resulted in a significant increase in beta CTX (RM ANOVA, p < 0.001; Tukey test, p < 0.05 for 60–180 min) with maximum increases of 64% and 80%, respectively. There were no significant differences between the AUCs for teriparatide injections and AUCs for EDTA infusions. During the control test period, there was a mild, but statistically significant decrease in serum beta CTX as early as at 60 min (RM ANOVA, p < 0.001; Tukey test, p < 0.05 for 60–180 min). There were significant differences between the AUCs for control test period and AUCs for teriparatide injections or EDTA infusions (ANOVA, p < 0.001).

Plasma N-MID osteocalcin and serum PINP, markers of bone formation, remained stable over 3 h of study period after both PTH and EDTA administrations in five studied men (data not shown).

## Discussion

We demonstrated that in healthy young men both the transient stimulation of endogenous PTH secretion and the single subcutaneous administration of teriparatide increased serum beta CTX concentration, the degradation product of mature collagen type I, which is released into the circulation during osteoclastic bone resorption. In accordance with known circadian variation in bone resorption, a mild but significant decrease in the serum beta CTX concentration was observed during the control test period. The rapid increase in the serum beta CTX concentration as early as 1 hour in response to PTH suggests that PTH affects the function of mature active osteoclasts. This is consistent with previous morphological evidence of changes in activity of resorbing osteoclasts within minutes after exposure to hormone (Holtrop et al. 1979; Miller, 1978).

The rapid increase in serum beta CTX concentrations seems to reflect the secretion activity of osteoclasts. Resorbing osteoclasts are dynamic cells with a high membrane turnover which have specific machinery for uptake and transcytosis of bone degradation products, such as type I collagen fragments (Nesbitt and Horton, 1997; Salo et al. 1997). Once resorption has started, endocytosis and transcytosis of the endocytosed material from the ruffled border of osteoclast are fast processes, which cover the speed requirements of the large membrane turnover during bone resorption (Stenbeck and Horton, 2004). Also, PTH may control transport processes of bone degradation products through the lining bone cell complex from the bone fluid compartment to the circulation (Talmage et al. 1976).

The osteoclast-mediated resorption may be involved in the initial phase of PTH anabolic action on bone (Onyia et al. 2000; Martin and Sims, 2005). Experimental data indicated that active osteoclasts are required within one hour after PTH administration for the full anabolic effect of PTH to be achieved (Gooi et al. 2006). The anabolic effect of PTH is blunted by treatment with bone resorption inhibitors such as bisphosphonates (Delmas et al. 1995; Wronski et al. 1993; Finkelstein et al. 2003). As expected, during the period of 3 hours there was no apparent effect of PTH administration on markers of bone formation at least in five studied men. A more prolonged period of obeservation should be needed to observe changes in these markers of bone formation.

The present study has several important limitations. Our subjects were healthy young men and the results do not allow conclusions concerning osteoporotic patients. Separate tests in this study were performed sequentially and in open label. We did not measure plasma kinetics of the amino terminal peptide (hPTH 1–34) after teriparatide administration; therefore it is not possible to compare the plasma profile of teriparatide with the endogenous PTH concentrations. However, it has been shown that teriparatide concentration peaks about 30 min after its single administration (Lindsay et al. 1993). The observed rapid change in serum beta CTX concentrations seems to reflect the secretion activity of osteoclasts. However, we did not measure directly osteoclast activity and our assumption is based primarily on previously published reports of animal experimentation.

In conclusion, single teriparatide administration as well as stimulation of endogenous PTH results in an acute increase in the bone resorption, as assessed by serum beta CTX. Our data support the concept that, in the initial rapid phase of PTH action on bone, osteoclast-mediated resorption is involved. Serum beta CTX seems to be a useful biochemical marker in the monitoring of acute effect of PTH on bone resorption.

## Figures and Tables

**Table 1:** Mean changes (1 SD) in serum beta CTX concentrations (ng/l) in response to EDTA infusions, teriparatide injections and control test period after a standard calcium load in 12 men.

Period (N = 12)	baseline	30 min	60 min	90 min	120 min	180 min
**Control**	441 (138)	389 (115)	376 (132)*	373 (158)*	356 (141)*	371(141)*
**Teriparatide**	349 (120)	319 (128)	458 (141)*	533 (152)*	554 (146)*	611 (186)*#
**EDTA**	330 (126)	333 (124)	436 (146)*	508 (147)*	510 (150)*	481 (134)*#

* p < 0.001 repeated measures ANOVA and p < 0.05 for comparison with baseline after adjustments for multiple comparisons (Tukey test).

# p < 0.001, ANOVA for comparison AUCs between teriparatide or EDTA and control test period.
